# Field serological investigation for peste des petits ruminants, foot-and-mouth disease, and bluetongue diseases in illegally introduced animals in Egypt

**DOI:** 10.14202/vetworld.2020.1661-1666

**Published:** 2020-08-21

**Authors:** Wafaa Abd El Wahab Hosny, Eman Mohamed Baheeg, Hala Abd El Raheem Aly, Samia Said Abd El Nabi, Nadia Maher Hanna

**Affiliations:** 1 ELISA Unit and Virus Strains Bank, Animal Health Research Institute, Agriculture Research Center, Dokki, Giza, Egypt; 2 Department of Virology, Animal Health Research Institute, Agriculture Research Center, Dokki, Giza, Egypt

**Keywords:** bluetongue and foot-and-mouth disease sheep-goats, competitive enzyme-linked immunosorbent assays, peste des petits ruminants

## Abstract

**Aim:**

In this study, laboratory scoping on the viruses that cause peste des petits ruminants (PPR), bluetongue (BT), and foot-and-mouth disease (FMD) was performed to evaluate the current status of animals illegally introduced into Egypt. This study aims to help control these infectious illnesses and tries to prevent the introduction of other strains of these three viruses to Egypt, as these illnesses spread quickly if not controlled.

**Materials and Methods:**

In the year 2018, 62 serum samples were collected and serologically tested through competitive enzyme-linked immunosorbent assays (ELISA) kits to detect antibodies against PPR, BT, and FMD, which are three important transboundary infectious illnesses.

**Results:**

The results indicated that 60 out of 62 serum samples were positive for PPR antibodies (96.7%), 31 out of 62 were positive for FMD antibodies (50%), and 59 out of 62 serum samples were positive for BT antibodies (95%).

**Conclusion:**

This study revealed that PPR, FMD, and BT can be introduced into Egypt through the illegal introduction of sheep and goat from neighboring countries. Laboratory diagnostic abilities should be improved for the early detection and control of these illnesses.

## Introduction

Viruses represent serious threats to animal health; therefore, the early and quick diagnosis and identification of viral pathogens are essential. The diagnosis of viral illnesses is necessary for determining control strategies and identifying the prevalence of viruses in different forms. Many viral pathogens such as the peste des petits ruminants virus (PPRV), bluetongue virus (BTV), and foot-and-mouth disease virus (FMDV) were among those that induce serious economic losses in the field [1]; consequently, control of these illnesses is essential, not only for reducing economic losses but also for increasing livestock production.

PPR is an important infectious viral disease that affects domestic animals and small, wild ruminants. This disease threatens food security and the sustainable livelihood of farmers across Africa, the Middle East, and Asia**.** This illness is emerging in new regions and is causing significant economic loss. The PPR virus belongs to the genus *Morbilli*, sub-family *Paramyxovirinae*, family *Paramyxoviridae*, and order *Mononegavirales* [2,3].

During PPR, the animals have fever (up to 41°C) throughout the acute stage of illness that lasts for 3-5 days. This could also be accompanied by depression, anorexia, and muzzle dryness. The animals also exhibit watery nasal and lachrymal discharges and erosive lesions shaped within the oral cavity that may become necrotic. In severe cases, these necrotic lesions progress to the appearance of a fibrin deposit (caseous deposit) on the tongue. In later stages of illness, animals develop diarrhea, cough, and abdominal breathing. Finally, the animal may become dyspneic, suffering from increasing weight loss and emaciation, finally resulting in death. In some cases, specifically during mild infections, animal health might improve, returning to its pre-infection health status within 10-15 days of infection. The morbidity rate may reach 100% with a high mortality rate during acute illness [4,5]. The afore-mentioned clinical signs and mortality may vary significantly depending on the virulence of the viral strain and the immunological state of the affected animal [6].

PPR infection is transmitted by contact between infected and susceptible animals or by oral or respiratory routes as the virus is present in high concentrations in lacrimal and oral secretions [7].

The diagnosis of the illness can be performed by detecting the serum antibodies directed against the N protein, since the *Morbillivirus* N protein is highly antigenic. The diagnostic enzyme-linked immunosorbent assays (ELISA) kits with high specificity and sensitivity that detect antibodies directed to either the N or H proteins of the virus, indicating seropositivity within a population, are available [8].

The BT Viral Illness (BTVD) is a *Culicoides*-borne illness that is recognized by the Organization of Animal Health (OIE). This disease affects ruminants, primarily sheep and is caused by BTV. BTV is an arbovirus, as well as an RNA virus belonging to the genus *Orbivirus* in the family *Reoviridae*. BTV transmission is facilitated by midges belonging to *Culicoides* species. It can also be transmitted by direct contact with some serotypes, as well as by vertical transmission from mother to fetus [9,10].

Clinical signs of BTVD are mainly attributable to vascular permeability and include fever, hyperemia and congestion, facial edema and hemorrhages, and erosion of mucus membranes. However, in mild illness, transitory hyperemia and slight ocular and nasal discharge may be observed [11]. In Egypt, the illness is generally mild in endogenous sheep since the classical symptoms of the illness are not commonly seen, so the detection of infected animals becomes difficult when based on clinical profiles [12].

BTV causes severe morbidity and mortality in sheep, whereas infection is sub-clinical in some domestic and wild ruminants. BTV genomes are composed of ten fragments of double-stranded RNA encoding seven structural (VP1-7) and four non-structural (NS1-4) proteins**.** It is classified into 27 serotypes based on the genetic and antigenic features of the neutralizing protein VP2. The VP7 protein could be a major determinant of serogroup specificity, and most of the serological assays that detect BTV are based on detecting anti-VP7 antibodies**.** BTV distribution has drastically changed in the last decades: Previously, it had been primarily distributed in tropical regions; however, since 1998, outbreaks in the Mediterranean to Northern Europe have been reported [9].

Competitive ELISA (c-ELISA) can measure BTV antibodies while not having the cross-reactivity problems encountered when performing the agar gel immune-diffusion test. This is extensively utilized in clinical laboratories for the detection of BTV antibodies and is sensitive, specific, and reliable [9].

Foot-and-mouth disease (FMD) is a severe and extremely contagious viral illness that primarily affects cloven-hoofed livestock, including cattle, sheep, goats, pigs, and water buffalo. It is caused by the FMDV [13], belonging to the genus *Aphthovirus* within the family *Picornaviridae*. There are seven immunologically distinct serotypes: A, O, C, SAT1, SAT2, SAT3, and Asia1 that do not confer cross-immunity [14]. FMD is endemic in parts of Asia, Africa, the Middle East, and South America. Whereas serotypes O and A are widely distributed, the SAT viruses are found mainly in Africa with periodic incursions into the Middle East and Asia, with one SAT virus currently only found in Asia [15].

FMD causes high economic losses due to the reduction of milk production, deaths in young animals, and reduction of animal weight gain. The severity of clinical signs varies according to the viral strain, exposure dose, age and breed of the animal, host species, and degree of host immunity. These signs vary from mild or unapparent to severe [16]. While morbidity may approach 100%, mortality, in general, is low in adult animals (1-5%), but is higher in young calves and piglets (20% or higher). Recovery in uncomplicated cases usually takes about 2 weeks [17].

Sheep play an important role in the transboundary spread of FMD [18]. They become carriers and act as an infection reservoir [19]. Animals could be carriers in three cases: After recovery, when they have the sub-clinical form of FMD, and when vaccinated animals are subjected to infection. The duration of the carrier state differs according to the species it infects, lasting for 5 years, 3 years, and 9 months in cattle, African buffaloes, and sheep and goats, respectively. On the other hand, deer and antelope can carry the virus for long periods [20].

The first outbreak of PPR in Egypt occurred in January 1987 in goats at Kafr Hakim, Embaba, Giza Governorate, wherein 30% of the animals had died. PPR virus was once isolated from Vero cells from lymph nodes and spleen and was recognized using the direct fluorescent antibody method (DFAT) [21]. More recently, an outbreak in 2006 in the Aswan Province once again highlighted the potential for infected goats to occasionally be asymptomatic, while others develop a severe medical disorder [22]. A study [23] also performed a genetic analysis of PPRV and found that on comparing the N gene sequence of both PPRV isolates, they saw that there is a homogenous population of PPR virus isolates of up to 99%. The PPRV isolates used in their study and all preceding Egyptian isolates belong to lineage IV in terms of phylogenetic analysis.

BTVD was reported for the first time in Egypt as early as 1974 [24]. The virus was isolated through VERO cells and from embryonated chicken egg (ECE). The virus was titered, and its serotype was determined to be 16. In addition, a BTV infection outbreak in imported Marino sheep had been previously documented by Gibbs and Greiner [25]. The reported BTV serotypes within the subsequent outbreaks were BTV-1, 4, 10, 12, and 16. In 1980, the BT clinical signs seemed to be more aggressive. The virus was discovered through the neutralization test (NT0 complement fixation test [CFT]) and the agar gel precipitation test (AGPT) [26]. In 1991, the virus was isolated from an animal slaughterhouse and propagated in a Baby Hamster cell (BHK-21) culture and ECE, and was, afterward, characterized through CFT and fluorescent antibody technique (FAT) [27], wherein it was found that the BTV was circulating in all ruminants. The virus was still circulating until it was isolated and identified in 2002 [28], in a study, wherein BTV was isolated, and a vaccine trial was applied using the isolated and known serotype 9. Another study [29] isolated and propagated the BT virus in Baby Hamster kidney cells (BHK-21) and was identified by ELISA, electron microscopy, and reverse transcription-polymerase chain reaction (RT-PCR). In another study, the presence of BTV among cattle that had been found BTV-seropositive through ELISA [30] was investigated. These cattle living in proximity to sheep and goats were previously found to be BTV-seropositive.

Several outbreaks of FMD have affected cattle, buffaloes, sheep, and goats in Egypt since the 1950s, with the FMD virus serotype O1 [31]. In 2006, clinical cases of FMD were recognized in cattle, and FMDV type (A) was determined [32]. In 2012, FMDV SAT2 was reported within cattle and buffaloes in six outbreaks in eight governorates [33]. This serotype (SAT2) was spread throughout Egypt, Libya, and Palestine. Phylogenetic analysis of the isolated serotype (SAT2) showed that circulating FMDV is genetically related to the serotype isolated from Sudan [34] between 2000 and 2010. A virus involved in a more recent outbreak belongs to the A/AFRICA/G-IV lineage, which was observed in Egypt in 2016; genetic studies discovered its relationship to the viruses collected from Nigeria in 2015 and from Cameroon in 2013 [14].

This study aims to help control PPR, BT and FMD and tries to prevent the introduction of other strains of these three viruses to Egypt, as these illnesses spread quickly if not controlled.

## Materials and Methods

### Ethical approval

Experiments were carried out in accordance with the guidelines laid down by the Institutional Animal Ethics Committee and in accordance with the local laws and regulations.

### Serum samples

In January 2018, 62 serum samples were collected from sheep that were illegally introduced from Sudan to the El-wady El-Gadid governorate in the southwestern boundaries of Egypt.

### ELISA kits

#### PPR c-ELISA kit

ID Vet Innovative Diagnostics, France: ID Screen PPR Competition. This kit was used for the detection of PPRV antibodies by c-ELISA. The assay was carried out according to the manufacturer’s instructions. The mean optical density (OD) value of the positive control (posC) was calculated to be <30% of the OD of the negative control (NgC), while the OD of NgC is greater than 0.7. A sample representing a S/N ratio of ≤50% is considered positive, those greater than 50% and ≤60% are considered doubtful, and those greater than 60% are considered as negative.

#### BT c-ELISA kit

ID Vet Innovative Diagnostics, France: ID Screen BT Competition. Serum samples were examined using this kit to detect antibodies against the BTV VP7 protein in sheep, goat, cattle buffalo, and deer serum or plasma samples.

#### FMDV c-ELISA kit

ID Vet Innovative Diagnostics, France: ID Screen FMDV Competition. Serum samples were examined using this kit for the detection of antibodies against the FMDV non-structural protein (NSP).

## Results and Discussion

The detection of PPR antibodies against the N protein, using a c-ELISA kit, revealed 60 out of 62 positive samples (96.7%) in sheep serum samples. The detection of BT antibodies against the VP7 protein resulted in 59 positive samples out of 62 sheep serum samples (95.1%). The detection of FMD antibodies against the non-structural protein resulted in 31 out of 62 positive samples from the total sheep serum samples (50%).

This study was applied on 62 small ruminants from the El-Wadi El-Gadid governorate in 2018; these animals were suspected to be previously exposed to PPR virus, BTV, and FMDV, which are three important transboundary illnesses.

The collected serum samples were examined by c-ELISA. PPR antibody detection showed that 60 out of 62 samples (96.7%) were positive. The highest proportion of seropositivity could be attributed to the illegal introduction of animals from Sudan; thus, animals such as sheep and goats, if imported legally, must be put in quarantine before entering the country. Furthermore, many factors increase animal susceptibility to PPRV, including young age, low maternal immunity intake, poor nutritional status, and drastic climatic conditions [1]. Moreover, in a study [35] of 110 serum samples from ill, recovered, and apparently healthy animals that were tested for the presence of PPR antibodies by c-ELISA, 84 animals were positive for PPR antibodies**.** Next, anti-PPRV antibodies were detected in sheep and goat sera samples using C-ELISA in 8/10 (80%) of the tested samples [36]. Another study by Nafea *et al*. [37] revealed that antibodies were detected in five sheep sera samples (5/7; 71.4%) using C-ELISA, and they stated that PPRV is still circulating in Egypt, leading to outbreaks in its major host (small ruminants); therefore, an effective PPR vaccination program is recommended to be regularly administered in Egypt along with strict quarantine measures at the borders to prevent the introduction of new PPRV genotypes [38]. Sudanese strain caused a PPR outbreak in Ismailia, Egypt in 2010 and 2012 [23].

The BTV serological response develops 7-14 days after infection, generating both neutralizing and non-neutralizing anti-BTV antibodies that are usually considered long-lasting. The diagnostic tests for BTV infection can, therefore, be based around the detection of these anti-BTV antibodies [9].

The current study used C-ELISA, which is a prescribed test in international trade that was developed to measure BTV-specific antibodies without detecting antibodies that cross-react to the orbiviruses [9]. In our study, BTV antibodies were detected in sheep sera (59 of 62/95.1%) (Table-1). Positive serum samples imply that BTV-specific antibodies, which are still circulating in the examined animals without detectable signs, are due to sub-clinical infections. This phenomenon occurs in places where BT is an endemic illness, but the virus may be of low virulence, and resistant species have a sufficiently high immunity level. The highest proportion of seropositivity may be attributed to the illegal introduction of animals from Sudan, from where the enzootic nature of BTV in large regions of the African continent was reported [39]. In addition, there were risks of windborne carriage of infected *Culicoides* from distant endemic areas [40]. A study showed that ELISA resulted in 98% sensitivity for the detection of BT in 200 serum samples collected from the El-Sharqyia, El-Daqahlyia, and El-Qalyoupiya governorates [29]. Moreover, a previous study [41] showed that out of 1028, serum samples from sheep, 15.7% tested positive for BT antibodies.

**Table-1:** Results of the detection of antibodies against PPR, FMD, and bluetongue viruses by c-ELISA assay.

Disease Ab	Species	Results			Total number of samples

		+ve	−ve	% of +ve	
PPR	Sheep	60	2	96.7	62
BT		59	3	95.1	62
FMD (Nsp)		31	31	50%	62

*The percentage represents samples only. PPR=Peste des Petits Ruminants, FMD=Foot-and-mouth disease, c-ELISA=Competitive enzyme-linked immunosorbent assays, BT=Bluetongue

The results of the ID screen FMDV competition ELISA test in this study proved the presence of antibodies against NSP of FMDV in the serum of samples obtained from the El-Wadi El-Gadid governorate that can be attributed to natural FMDV infection, with a positive percentage of 50%. Part of the vaccinated animals may be sub-clinically infected if they are subsequently exposed to the homologous virus, and can, therefore, transmit infection for up to 14 days after vaccination, even when they become immune to the development of clinical illness [42]. Seroprevalence against FMDV was recorded in sheep and goats and enabled the elucidation of the potential role of small ruminants in FMD epidemiology [43]. Serum samples were tested for NSP antibodies using NSP ELISA, which is an assay that is simple to perform and suitable for large scale serological surveillance. It can also differentiate between vaccinated and infected animals. In addition, it can detect 3ABC antibodies, which have a higher concentration in the serum compared to others. In addition, 3ABC ELISA has already been used in ruminants as a DIVA (Differentiation between infected and vaccinated animals). It was also indicated that FMDV activity in small ruminants was reported as seroconversion. This indicates that small ruminants, when kept in close contact with large ruminants, could serve as reservoirs of FMD virus and as potential sources of infection for susceptible livestock. Results of detection of FMDV and PPRV antibodies using the OD of the ID Vet c-ELISA Kit and the ID screen PPR Competition ELISA Kit, respectively, indicated that testing of 50 serum samples from the Marino sheep flock resulted in 38/50 (76%) and 32/50 (64%) positive for antibodies against FMDV and PPRV, respectively [1]. The study reflected the high susceptibility of the imported flock to infection with FMDV and PPRV, which are viruses that are endemic in the Kingdom of Saudi Arabia; as such, so the flocks imported for slaughter should be quarantined and vaccinated. This condition is mostly found in young animals or after mixing animals from different origins. Mahmoud *et al*. [44] declared that their work aimed to elucidate the different methods for diagnosis and control of FMD that affect small ruminants involved in dairy production, namely, sheep and goats. Sheep and goats play a role in FMD epidemiology, as they become carriers and act as reservoirs of infection. Detecting antibodies against the non-structural proteins (NSPs) of FMD using indirect-ELISA were successful for DIVA, which is greatly important in managing FMD.

## Conclusion

Our study shows that PPR, FMD, and BT can be introduced into Egypt through the illegal transport of sheep and goat from neighboring countries. Restrictions for animal movement and quarantine should be applied to prevent the incidence of disease spread and outbreaks. Until now, there are no accessible DIVA tests for PPRV detection; therefore, there is a need for DIVA diagnostic tests and marker PPR vaccines to differentiate between field wild-type and vaccine PPRV strains. Quarantine of affected animals and elimination of contact fomites, as well as disinfection of the affected premises, are, therefore, recommended to mitigate disease spread.

## Authors’ Contributions

WAH, EMB, HAR, SSA, and NMH conceived the study, carried out the laboratory work, and analyzed the data. WAH supervised the research. EMB and HAR drafted the manuscript. All authors read and approved the final manuscript.

## Competing Interests

The authors declare that they have no competing interests.

## Publisher’s Note

Veterinary World remains neutral with regard to jurisdictional claims in published institutional affiliation.
